# Ten Testable Properties of Consciousness

**DOI:** 10.3389/fpsyg.2020.01144

**Published:** 2020-06-24

**Authors:** Christopher W. Tyler

**Affiliations:** California Institute of Integral Studies, San Francisco, CA, United States

**Keywords:** consciousness, brain, neural substrate, hard problem, properties, emergence, empirical evidence

## Abstract

This article develops a view of consciousness in the context of a new philosophical approach that invokes the concept of emergence, through which the operative principles of each level of organization of physical energy flow are functionally dissociated from those of the levels below it, despite the continuity of the physical laws that govern them. The particular form of emergence that is the focus of the present analysis is the emergence of conscious mental processing from neural activity carried by the underlying biochemical principles of brain organization. Within this framework, a process model of consciousness is developed to account for many of the experienced aspects of consciousness, many that are rarely considered in the philosophical discourse. Each of these aspects is rigorously specified in terms of its definable properties. It is then analyzed in terms of specific empirical tests that can be used to determine its neural substrate and relevant data that implement such tests. The article concludes with an analysis of the evolutionary function of consciousness, and a critique of the Integrated Information Theory approach to defining its properties.

## Introduction

### Philosophical Background: Principles of Functional Emergence

Before addressing the properties of consciousness, it needs to be placed in the context of the overall physical reality from which it emerges. This is conceptualized in the form of Emergent Aspect Dualism (Tyler, 2015, 2018, 2019), which reconciles the epistemic dichotomies of monism and dualism, energy and matter, emergence and continuity, neural activity and consciousness, free will and determinism, and even continuous reality with the superposition and multiple worlds interpretations of quantum physics. This philosophical approach takes the view that complex levels of organization of physical energy are both ontologically and functionally emergent from more basic levels (physical energy being defined as the flow of some physical substance, or stored propensity to flow in the case of potential energy^1^). Thus, the forms of energy at play at the level of subatomic physics are kinetic and potential energy, as captured in the continuous Schrödinger Equation. Indeed, the best-known equation in physics is the Einstein Energy Equation, E = mc^2^, which expresses unity of energy and matter and, in stellar evolution, the emergence of matter from the raw energy of the Big Bang. It is emphasized that the initial references should be consulted for detail of this contextual overview.

As is well recognized, the emergence principle gives rise to the organizational hierarchy adumbrated as consisting of quantum physics, particle physics, macro physics, physical chemistry, biochemistry, cellular biology, neurobiology, neurophysiology, systems biology, and the psychology and philosophy of consciousness. At the level of biology, the organization of cells such as neurons is emergent from the continuity of their component proteins, one major example being the enclosing property of a cell membrane supporting the maintenance of life that is lost when the integrity of the membrane is punctured. The support function of the cells thus emergent from the enclosure of the membrane, a physical instantiation of the Gestalt principle of closure that provides the critical life-sustaining property of a segregated internal environment. This concept of emergence transcends the strong/weak distinction that has become embedded in the philosophy of emergence (e.g., Hartmann et al., 2019; Turkheimer et al., 2019), since it is “weak” in the sense that it is built up step-by-step from its elementary constituents, but “strong” in the sense that an entirely new principle of operation emerges once closure is achieved (Tyler, 2018).

The most immediate form of emergence specific to the human organism is the emergence of consciousness^2^, which is the main focus of the present analysis. Specifically, the evidence from our general experience of human mortality, and from neurosurgery in particular, supports the concept that consciousness is an emergent property of the physical activity of the neurons of the brain. In the general conception, this activity is carried by neurotransmitters such as glutamate at the input to neurons and by sodium/potassium ion exchange along their output connections, so these are the physical substrate of the neural activity in question (which ultimately is itself an emergent form of energy flow of the underlying subatomic processes). It is not intended to imply that consciousness is a form of energy *per se*, but a particular form of *organization* of the energy flow of such neural activity.

The substrate of consciousness is assumed to be the neural activity of the brain, but what makes consciousness unique is that it is the only process that “we” know from the internal perspective of what it is like to ***be*** that process (a different form of internal exclusivity). It is this emergent *internal* perspective that entails the hard dualism of the “Hard Problem” of consciousness (Chalmers, 1997), since we cannot take the internal perspective of anything beyond our own brain process (and our own brain process is the specific one in which our consciousness is obligatorily embedded as our internal viewpoint on sensory and working memory information). Thus it is the privacy or exclusivity of our brain process to our own personal subjectivity that entails a dualism that is emergent from fundamentally monistic complex energy function (Hasker, 1999; Tyler, 2018), as opposed to the classic Cartesian dualism of the separate realms of mental and physical substances.

The final dual aspect of this philosophy is the unity of free will and determinism. The primary assumption of the philosophy is that brain function is fundamentally deterministic (though the product of such a complex system that much of the activity is effectively indeterminate noise). However, as MacKay (1960) has shown, even if an all-knowing external system (or “God”) had access to all the information in your^3^ brain to predict the next optional decision, it could not convey the information to your subjective decision-making capability, or “will,” in a form that could necessarily enforce the decision. You would always have the freedom to decide not to follow the all-knowing prediction once it was presented to you. Thus, in your subjective experience, you always have to deliberately make each decision, or deliberately decide to leave the decision to someone or something else. Your free will is inherent, and cannot be removed by an external predictor, no matter how well-informed it is, even though the entire process is, by assumption, fully determinate at the physical level.

MacKay’s paradigm resolves these aspects of free will by illustrating that there is no external prediction that necessarily holds force over the internal decision – the process is always subject to a further decision (although the prediction may provide a helpful weighting of the pre-decision factors). And since life involves a continuous series of decisions made by the most complex organ known to nature, it is hard to imagine that the inherent noise variations throughout the sequence of decisions could be considered deterministic throughout a human life.

This analysis might raise the issue of how this paradigm would apply to a non-conscious automaton programmed to make decisions based on accumulated evidence, which could be programmed to process an external prediction as part of its decision inputs (and respond in a way that is not previously predictable). Stating it in this form makes it clear that the issue of free will is separate and independent of the presence of consciousness *per se*, and highlights the question of what the core issue of free will actually is. Is it the question of whether your entire life is laid out (as part of “God’s plan”), without you having the power to affect it? Or whether you could in principle access a source that can inform you of the outcome of each decision you have to make in life, to avoid the challenge of having to struggle through the decision process? Although we humans are only recently developing automata with these kinds of capabilities, the same issues could be formulated for such non-conscious systems, although they only seem to be meaningful when viewed from the *internal* perspective.

This brief overview thus outlines how the Emergent Aspect Dualism philosophy simultaneously reconciles monism with dualism, energy with matter, continuous reality with superposition/multiple worlds, emergence with continuity, neural activity with consciousness, and free will with determinism.

### The Role of Consciousness in Quantum Physics

Returning to the physical underpinnings of neural activity, quantal events are treated in standard quantum theory as being in a probabilistic superposition of physical states in which multiple outcomes coexist until an observation made (Feynman, 1985). Emergent Aspect Dualism, on the other hand, treats all these potential outcomes as existing solely in the mental space of the observer (and in the communications of the physics community by whom the probabilities are calculated), but only *one* outcome as having occurred in the underlying reality (Minev et al., 2019). Thus, the unconventional position of Emergent Aspect Dualism is that the many worlds of the resultant outcomes, and their probabilistic superposition, exist only in the mind (or its computational extensions), not in the quantum reality as generally understood^4^.

In this way, the emergent dualism that is the outcome of a sufficiently complex process such as the human (or other) brain lies at the heart of the paradoxes of probabilistic quantum physics that supposedly give rise to it. However, applying this philosophical framework to the role of consciousness in quantum physics leads to the conclusion that probability is not a concept that is inherent in physical processes, but an *analytic concept of a human mind* with the memory to accumulate repeated instances of physical events. It is an inherent property of probability that, by definition, it incorporates multiple defined outcomes (of **p** and **not p**, for example) and associates each one with a weight (necessarily based on past experience with those outcomes). These multiple, or complementary, outcomes are therefore in a state of **conceptual superposition** within the specification of probability, *per se*. Since probability, as a mental concept, inherently embeds the superposition of the complementary states, it follows that the Schrödinger Cat paradox and the collapse of the wavefunction are resolved by realizing that the superposition is a property of the mental representation rather than of the physical reality (Schrödinger, 1935; Tyler, 2015, 2019). It should be noted that this clarification of the relationship between consciousness and the properties of the quantum realm is included as an antidote to the widely disseminated concept that the properties of consciousness could derive from the putative non-classical properties of the quantum realm (Wigner, 1970; Penrose, 1995; Bohm, 2002). Nevertheless, the following analysis of the properties of consciousness in terms of classical biochemical processes does not depend in the resolution of that controversy.

### A Definition of Consciousness

It is well in developing an analysis of a phenomenon to attempt a definition of the subject matter under investigation. The present treatment is focused on what Block (1995) terms “phenomenal consciousness,” the direct experience of being vividly aware of the flow of events (as contrasted with “access consciousness,” which corresponds to the information content of mental operations controlling behavior, as in the Integrated Information Theory of Tononi, 2008). Searle (1990) provides an operational definition of phenomenal consciousness as follows; “By consciousness I simply mean those subjective states of awareness or sentience that begin when one wakes in the morning and continue throughout the period that one is awake until one falls into a dreamless sleep, into a coma, or dies or is otherwise, as they say, unconscious.” I would extend the wake/sleep distinction for the consciousness definition here to incorporate the distinction of “working memory” (Baddeley and Hitch, 1974), or operational thought, in that consciousness is “what it is like” (Nagel, 1974) to imagine or think about sensory or memory contents at a given moment (as contrasted with all the possible things in memory that we could be thinking about but are presently out of awareness). This contrast marks a major distinction between to the direct operations of phenomenal thought itself, as opposed to the neural organization that is available to contribute to thought (similar to Block’s “access consciousness”). All definitions of consciousness are ultimately either ostensive or tautological, but it is hoped that these descriptions help to define the matter at hand relative to the reader’s own experience.

Nevertheless, Block’s (1995) concept of access consciousness apparently allows that it can in principle exist without incurring phenomenal consciousness. In this sense, it would constitute a form of ***un***conscious information processing that is not distinguishable from what could occur in a (biological or computational) neural network. Indeed, Block’s access consciousness thus reads as very close to the operational concept of “working memory,” the set of conceptual processes that control speech and behavior. As such, they can be investigated empirically without reference to the conscious experience of the individual under investigation. The present treatment, on the other hand, is focused on the basic set of phenomenally experienced properties of consciousness (which are necessarily those of the author as a consequence of the privacy restriction, but laid out in a form that it is hoped will resonate with the experience of the reader). The basic properties under consideration in the following are defined purely phenomenologically, without reference to the neural properties of the brain at any level of investigation.

## Analysis

### The Nature of Consciousness

What, then, is the nature of the process of emergent consciousness (C^∗^) that is so characteristic of human experience? In overview, this article will focus on the following ten properties of phenomenal consciousness, providing specific examples of empirically definable tests for the **neural substrate for conscious processing** (NSCP^5^), including some classic and some less-recognized properties of consciousness. The concept of the NSCP is distinguishable from the long-established one of the NCC (the neural correlate of consciousness; Crick and Koch, 1990) in that many forms of brain processing can *correlate* with the properties of consciousness without necessarily forming its true neural substrate. It should be noted that the defining property of phenomenal consciousness, its phenomenality (or qualia, in the plural), is not included in the list because it is not clear how it could be testable. The ten NSCP properties are:

1. **Privacy.** The obvious NSCP basis of the privacy of individual experience is its derivation from the separate brain of each individual experiencing C^∗^.
2. **Unity.** Having unified or correlated activity: if a particular set of brain structures is identified as the NSCP, then they should show unitary activity when C^∗^ is reportable.
3. **Interrogacy.** One aspect of brain function that has not been investigated is ability to formulate questions, or interrogacy, which seems unique to a conscious mind.
4. **Extinguishability.** The NSCP must exhibit the same time course of complete extinction as does C^∗^ itself every time we fall asleep or are anesthetized, and be rekindled when we awake.
5. **Iterativity.** Any plausible NSCP measure must exhibit the iterative cycling through similar states of conscious experience over the experiential range of time scales.
6. **Operationality.** The operationality of working memory is a functional property of the NSCP that is readily accessible to techniques such as behavioral assessment and brain imaging.
7. **Multifacetedness.** Though unitary in its dynamics, the NSCP should exhibit the multifacetedness of the conscious qualia of the sensory field that is characteristic of C^∗^.
8. **Complex interconnectivity.** To be explanatory the NSCP should match the variety of multilevel interconnectivity of conscious experience.
9. **Autosuppressivity.** The attentional suppression that keeps C^∗^ moving on from each identifiable mental state to the next is a further property that can be identified in candidate mechanisms for the NSCP.
10. **Self-referentiability.** Human C^∗^ has the capability of representing itself within itself, so its substrate has to be able to exhibit the corresponding capability.

**FIGURE 1:**
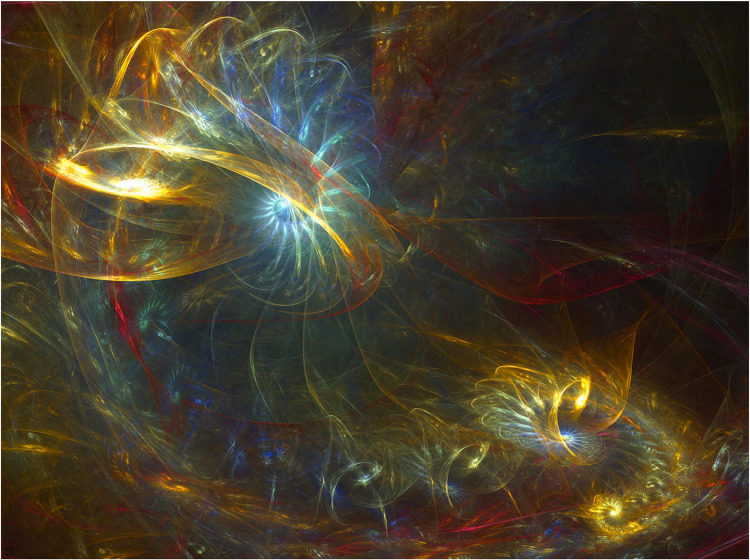
A complexly interconnected iterative and yet unitary structure that represents a dimensional implementation that captures the conceptual nature of the flow of consciousness (C*). Note that the coloration can be viewed as a stand-in for the qualia of the various contents of consciousness. (Free download of colorful-light-swirls-21710.jpg).

These ten properties may be explicated as follows:

1. **Privacy:** One of the irreducible properties of C^∗^ is its **privacy**. *Pace* science fiction, as far as we know, there is no way to share our individual C^∗^ with anyone else. Verbal and non-verbal forms of communication provide effective means of generating the illusion of sharing C^∗^, but (as too many lovers have found to their cost!), this is only a superficial level of apparent sharing, not a direct experience of another’s true internal experience. To meet this criterion, the NSCP must be brain-compatible and must not allow for direct interbrain communication. In the context of quantal theories of C^∗^, this means that the NSCP must not be based on any non-local quantal effects. (Those who accept the non-locality of some superordinate cosmic C^∗^ will, however, draw the opposite conclusion).
  Empirically, the privacy of C^∗^ is what for a long period was assumed to prohibit meaningful approaches to testing its properties. In recent years, however, the consistency of reports over time and across individuals has been taken to provide sufficient support for its meaningful investigation.^6^ For any group of individuals who agree that their internal experience does manifest one of the properties specified here, that property is testable, while ascertaining the proportion that do not agree is an empirical specification of the prevalence that property in the population.
  A standard objection to the communicability of a private experience is Wittgenstein’s (1953) argument that a private language would be incommunicable (§256–§271). Here, on the other hand, it is argued that this issue is largely addressed by the ostensive nature of language learning – we develop common concrete concepts by pointing at external examples of them in the external world that are mutually available to the senses across individuals, and build up more abstract concepts by analogy from the concrete examples. The concept of consciousness, likewise, can be communicated by ostensive reference to the difference in mental experience between being awake and being asleep (without dreaming). (Likewise, dreaming *per se* is another private experience, but no-one questions the linguistic communicability of the concept of dreaming).
2. **Unity:** Under ordinary conditions, C^∗^ is experienced as **unitary** at a given moment. We have one experience at a time, although we may be able to rapidly switch among multiple experiences over short time intervals. The NSCP must, accordingly, occur either in a single brain site or in a unified neural net of some kind in the brain, rather than in multiple independent brain sites. [Note that neurological split-brain cases are a counterexample that require special treatment based on whether their conscious experience is, in fact, continuously dual or has some other organization (see Gazzaniga, 1985), but this medically instigated controversy will not be addressed here].
3. **Interrogacy:** Though not widely recognized, a defining property of C^∗^ is the ability to generate **questions** and represent potential answers. Complex systems other than the brain, such as galaxies, biological organs and the Internet, incorporate extensive recursive interactions and consist of energy processes that undergo development and evolution comparable to those in the brain. Although these systems can be said to process information, however, they cannot meaningfully be said to ask questions. It seems to be a unique property of a conscious system to formulate questions, and a function that gets switched on in humans at about the age of a year. This capability also entails (though perhaps not until a later age) the ability to envisage possible answers in an indeterminate superposition of their probabilistic states of likelihood.
4. **Extinguishability:** A primary property of C^∗^ is its lack of continuity. As emphasized by Searle (1990; see “Introduction”), it is **extinguished** every time we fall asleep, are anesthetized or a knocked out by a physical trauma, and is rekindled when we awake (and also, in a somewhat restricted form, when we dream). Although these states are deeply subjective, they can be attested in the form of memory markers of external events (such as our last memory of a radio program before dropping off to sleep). Dreaming is well established as being objectively indexed by rapid eye movements while asleep (REM sleep) and also being extinguished as we awake.^7^
5. **Iterativity:** Another well-established property of C^∗^ is its tendency to **iterate repeatedly** through similar states, both when there are problems to be solved (such as anxiously reiterating a worrying scenario) and as a form of pleasure (as in music, which reflects the consciousness of its listeners, and has continual repeats of phrases, themes and whole pieces over a wide range of time scales). C^∗^ is thus not a state *per se*, but an iterative sequence of repeated sub-processes, each often entailing a resonance with previous ones.
6. **Operationality:** The operational property is captured by the term “working” in the cognitive science concept of “**working memory**.” In other words, “memory” is the ability to store representations of aspects of the world as stable brain states, while “working” is the functionality of not only bringing them to C^∗^ but using them to answer questions either in relation to a single memory (first-order) or in relation to the relationships between memories (second-order). The remarkable property of such memories is that we are not conscious of the millions of memories that are maintained in a non-conscious state most of the time. We only become conscious of them when they are accessed by C^∗^ for a brief time, which roughly corresponds to the Theater of C^∗^ of Baars (1983). There seem to be two forms of access, one being first-order *inoperative* or factual access that is usually included in the functional usage of working memory without involving any operational changes to the stored information (e.g., “what country is Stockholm in?”), and the other being the second-order *operative* access that is well-described as “working memory,” to perform some operation on the stored information (e.g., “does a rotated q become a b or a d?”).
7. **Multifacetedness:** C^∗^ by its nature incorporates all **varieties of human experience**, from logical thought processes and imaginary journey planning through the irreducible qualia of direct sensory and indirect imagery experiences to the array of emotional experiences and primary internal states of C^∗^ such as pain and orgasm. Although we still may not be able to envisage what it would mean for the NSCP to exhibit, or possess, such experiences, it is a core requirement of the theory that it would be able to do so. At least in the case of thought or journey planning, the NSCP should be able to exhibit the activation of the specific memory states representing the sequential stages of the specific thought or journey in question.
8. **Complex interconnectivity:** C^∗^ is experienced as complexly interconnected, in the sense that each instantaneous state can proceed along many “lateral thinking” paths from any one state to many others (see Figure 1). Thus, while the concept of multifacetedness refers to the array of experiential states of C^∗^, interconnectivity refers to the transitional probability among and between these states. This **interconnected flexibility** is part of its generative or creative power. It is not like a finite state machine, that typical proceeds sequentially from any one state to a definite following state. C^∗^ is capable of exhibiting multiple connectivity from any facet to many other facets of human thoughts and feelings, unconstrained by logic. (Of course, in some cases well-trodden paths of thought do become established such that C^∗^ does operate analogously to a logical finite-state machine, but this may be more the exception than the rule). This property corresponds to the “global workspace” concept of Baars (1983).

9. **Autosuppressivity:** One of the sources of the variety and creativity of C^∗^ is that it tends to exhibit the property of burning out at any one state, **suppressing** the tendency to return to that state, thus impelling continuous movement to novelty. This is a well-known property of attention across the visual field (“inhibition of return” Posner and Cohen, 1984; Müller and Kleinschmidt, 2007), and is also a rule of a good writing style, to avoid using the same term or phrase repeatedly in a text. Indeed, this is the opposite of the behavior of a classical finite-state machine, which repeatedly follows the same path from any given state. Autosuppressivity is thus a major contributor to the creativity of humans and other organisms, though it may be overridden by the iterativity property, the tendency to stay in the comfort zone of the same sequential paths of behavior.
10. **Self-referentiality:** A final property of C^∗^ is its ability to **represent itself** as a component of the conscious field. This property harks back to Russell’s Paradox as a seemingly impossible feat: what is the set of entities that includes itself as a member? But this is a common experience, that we can be (acutely!) aware of ourself as a participant in the field of C^∗^. This property goes beyond the primary quality of the external referentiality of C^∗^, that it has the inherent quality of referring to some form of object outside itself (or what philosophers misleadingly term “intentionality”). C^∗^ is experienced as the continuous journey of an identified self, or ego, through the succession of states of experience; that is, not simply an undifferentiated stream of consciousness, but a series of actions and experiences from the viewpoint of an internal entity identified as “me.”

## Empirical Testing for the NSCP

**FIGURE 2:**
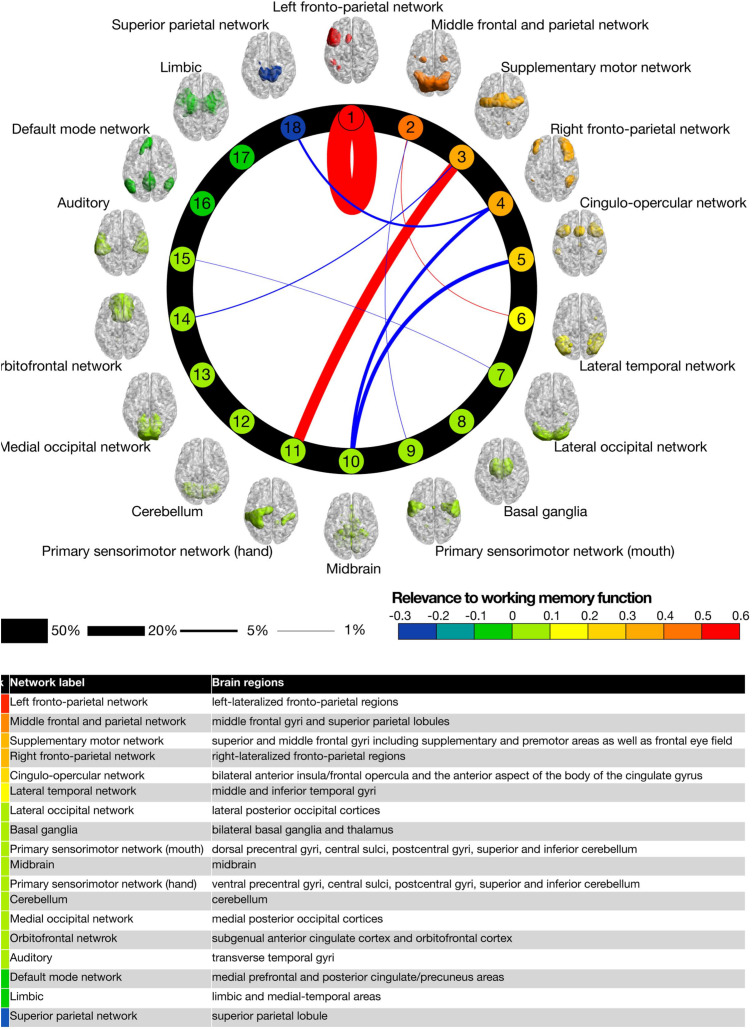
The intrinsic connectivity study of Yamashita et al. (2015), showing the strength of functional connectivities among 18 brain networks during a working memory task.

A plausible underlying assumption of the NSCP is that it must have a spatiotemporal isomorphism with the experiential properties of C^∗^. Thus, a core goal in specifying the above properties of C^∗^ is to define their spatiotemporal morphology in a testable form. Specific examples of empirically definable tests for the actual NSCP of the properties of C^∗^ specified above are as follows:

1. **Privacy.** The obvious basis of the privacy of individual experience is the separation of the brains of each individual. While this test is passed for the typical human brain configuration, it is not easy to set up the converse case, of co-extensive brains for non-private experience. An aspect that relates to this issue, however, is the correspondence between brain states for *comparable* experiences across individuals. When people judge that they have similar individual experiences in particular situations, these similar experiences should be expected to have similar NSCPs in terms of the recordable patterns of neural activity. An experiment along these lines was conducted at the Chinese Normal University of Beijing, in which a group of interacting individuals had their brain activity recorded simultaneously by functional Near-Infrared Spectroscopy (fNIRS) recording (Duan et al., 2015). It could thus be determined if their mutual brain activation patterns were more similar than when recorded asynchronously during non-shared experiences. The non-privacy of the individual brain processes would be validated if there was a relationship among the brain signals corresponding to their mutually experienced thoughts, when sensory communication was eliminated. Conversely, it is difficult to prove privacy, as it would require a null result from all possible forms of non-sensory communication. One approach is to review the history of studies of extra-sensory perception (ESP), which have all been shown to be a result of manipulation or fraud, despite the best intentions of many of the experimenters (Charpak et al., 2004). Even if a small amount of transmission could be validated under some rare circumstances, historically it has always been found to be such a minute proportion that it confirms the essential privacy of conscious experience under most circumstances.
  Empirically, a huge number of studies in human neuroscience are now identifying aspects of brain function in relation to a vast array of stimulation and endogenous environmental conditions. Many such studies, as exemplified by the “mind-reading” study of Huth et al. (2017), relate the brain activity to the reported contents of the individuals’ conscious awareness of a defined set of images. Although the accuracy, or information transfer rate, of these studies is low, they do represent a level of external access to the contents of consciousness, suggesting that sufficiently advanced technical system could breach the supposed privacy of consciousness to read minds. However, all such techniques rely on the veracity of the participants’ report of their conscious experiences, so their privacy is metained in principle if they choose not to cooperate in such investigations.
2. **Unity.** Consciousness is generally reported to be unitary at any given instant of time. For a particular set of brain structures to be identified as the NSCP, they should either be structurally unitary (such as the anatomical neural net of the claustrum) or have demonstrably correlated (i.e., unitary) activity across the multiple anatomical structures when C^∗^ is reportable. To pass this test, the correlation across structures should account for all, or a large proportion of, their recordable activation above the noise level of the recording technique, not just a weak correlation. If the NSCP has a unified *neural substrate*, that substrate should meet the criterion of showing uniform activity as the activity representing the different types of processing fluctuates elsewhere in the brain. If, on the other hand, the NSCP is represented by a particular form of *neural activity* (such as gamma-band energy), that form of activity should be manifested in each of the individual brain areas (cortical or subcortical) at the times identifiable as when the corresponding processing is occurring.
  A key issue arises in terms of the contents of C^∗^, which may switch rapidly over different topics over short periods of time, as in the example of a mother looking out for her children while cooking a meal and mentally preparing for a meeting with an upcoming legal client, with the TV news in the background. If a given individual reports that their C^∗^ is literally non-unitary, the corresponding non-unitary switches in activation of the putative NSCP should be identifiable by temporal correlation techniques.
3. **Interrogacy.** One aspect of brain function that does not seem to have been investigated either philosophically or empirically is the process of formulating questions, or interrogacy. Coming up with questions is a creative process that all philosophers and scientists engage in professionally, yet it does not seem to have been codified as a psychological process or studied in a neuroimaging context. Although question-generation is an established field of study in the educational field (Davey and McBride, 1986; Rosenshine et al., 1996), it has yet to become a topic of investigation the domain of cognitive neuroscience.
  The first requirement is to develop a protocol for putting an individual in a controlled state of question generation. Participants would be asked to think of a question about some topic that they have not previously formulated, and indicate when they have come up with a completed formulation. The panoply of brain imaging techniques can then be brought to bear on the issue of the particular substrate of the question-generation component of C^∗^, based on the time period immediately preceding the question-generation completion time. The NSCP should be coextensive with the brain processes underlying the interrogacy activity, once it is studied.
4. **Extinguishability.** The NSCP must exhibit the same time course of complete extinction as C^∗^ itself every time we fall asleep or are anesthetized, and be rekindled when we awake. This association could be tested with a button monitor that has to be held down while we are falling asleep or being anesthetized, but will be released by the muscular relaxation with the onset of the sleep state. In humans, this would be most easily tested with continuous scalp electroencephalography (EEG) recording but could be attempted with functional Magnetic Resonance Imaging (fMRI).
  Note that sleep research has long established the psychophysiological parellels between reported sleep states and EEG signatures. These show that the deep sleep associated with delta-wave activity (1–3 Hz) typically has little or no reportable conscious experience (Dement and Wolpert, 1958). The more rapid EEG activity normally associated with non-sleeping states qualifies as an NCC for C^∗^. This result, however, illustrates the difference between an NCC and a NSCP, since the absence of delta waves does not qualify as a substrate despite correlating with positive C^∗^, and the remaining EEG activity does not switch off during delta wave sleep.
  A prime example of empirical use of the extinguishability criterion is a study by Koubeissi et al. (2014), in which they found that electrical stimulation of the (left) **claustrum** above a certain threshold reversibly extinguished the participant’s C^∗^ for the time period of the stimulation, whereas corresponding stimulation of nearby brain regions had no such effect. This result suggests that the claustrum is an important component of the NSCP, which must therefore have a spatially localized substrate at least including the claustrum. (They did not have access to the right claustrum, so the respective roles of its two hemispheric partitions is undetermined).
5. **Iterativity.** Any plausible NSCP measure must exhibit the iterativity of repeated conscious experiences over the experiential range of time scales. This was the case for the electrical stimulation of the temporal lobe by Penfield (1958), where same long-forgotten conscious memory sequence was repeatedly evoked by stimulation at a single site in the temporal lobe (but not any other part of the brain). Without stimulation, the iterativity could be assessed by looking for long-range correlations within EEG or fMRI signals from memory areas, such as the temporal lobe. The intrinsic signal can be segmented into sliding segments each correlated with the next. Then the process is repeated at different scales of segment length. If any two segments show a significant correlation, this signal segment is then correlated throughout the signal duration to look for further repeats. In this way regular iterative patterns at a range of timescales can be uncovered.
6. **Operationality.** Operationality is the “working” aspect of working memory, the functionality of not only bringing relevant memories to C^∗^ but using them to answer questions either in relation to a single memory or in relation to the relationships between memories. This functional property is readily accessible to empirical techniques such as fMRI. Various NSCP brain sites associated with working memory have been identified through a vast range fMRI studies, but their specific roles and dynamics in relation to the operational properties of **experiential** working memory are not well understood. A particular example is the neural connectivity study of Yamashita et al. (2015), which assessed the intrinsic connectivity among 18 previously identified brain networks during learning of a three-back working-memory task: Is the current image the same as the one three-images back in the sequence? (which would be an example of a first-order question asked in relation to each individual memory). The performance improvement in this task was almost entirely attributable to the self-interaction of the dorsolateral prefrontal network (out of 171 possible network connections), with a few other weak contributions such as between primary and secondary motor cortex (see Figure 2). This result gives interesting insight into the improvement of operationality (and hence into the operationality *per se*) of working memory, with the effects localized to a particular cortical region that has been strongly associated with working memory in past studies.

The operational property can be tested either behaviorally or physiologically by using a behavioral task that requires accessing an operational relationship between two previously unrelated memories. A prime example of such operations is the mathematical task of performing an arithmetic operation on two numbers of a form that is not pre-encoded by mathematical tables (i.e., not memorized). The participant thus has to perform the operation of a real-time calculation to solve the task, requiring the retention of the numbers and manipulating the intermediate solutions in working memory to complete the calculation (Metcalfe et al., 2013).
7. **Multifacetedness.** The test for the property of multifacetedness is that the neural activity proposed as the NSCP for C^∗^ should be activated for all the multifaceted aspects of experiential consciousness. The testability criterion would be that any measurable neural process identified as the NSCP would be concurrent with one such experience, and *vice versa*, with no significant misses or false alarms in the coupling instances. One form of such multifacetedness is provided by the network interaction study of Yamashita et al. (2015; Figure 2). The 18 specified networks each have a defined function in the mental C^∗^ lexicon, although only a few are identified by the authors. In any particular task, several or many of these networks may be expected to be activated, with this activation representing the degree of multifacetedness of the C^∗^ experience. The relation of the identified functions of the activated networks to the subjective reports of the performers of such tasks can provide an index of the degree to which the fMRI activations represent the NSCP. To avoid reporting bias, it may be necessary to provide the reporters with a list of possible functions corresponding to the brain networks, for them to assess the degree of expression of each of them (and others not on the list).
8. **Complex interconnectivity.** Any one aspect of consciousness, such as awareness of a face, is not a simple state but a multilevel complex of experiential components from the basic “raw feel” to the communicative socio-emotional implications. To be explanatory, the NSCP should exhibit a similar variety of interconnectivity. A probe for such interconnectivity is provided by the network interaction study of Yamashita et al. (2015; see Figure 2). Although they are termed “networks,” most are dominated by one or a few cortical areas (in a 21st century manifestation of localization of function). Of the 171 possible connections, Yamashita et al. (2015) report strong connectivity among only nine of them (5%), predominantly motor networks, and negative associations between these networks and the Default Mode Network (DMN), whose function seems to be non-task-related personal reverie and planning. Thus, at this level of network analysis there is complex interconnectivity *within* each individual network, but relative isolation (specialization of function) *among* the networks.
9. **Autosuppressivity.** Once again, the attentional autosuppressivity that keeps C^∗^ moving on from each identifiable state to the next is a further property that can be identified in candidate mechanisms for the NSCP. This property is already well-substantiated as “inhibition of return” in attentional and saccadic target selection in visual search (Posner and Cohen, 1984; Rafal et al., 1989). The issue with such studies is that they do not index C^∗^ directly, so the inhibition of return is not necessarily associated with C^∗^ *per se*, in both directions of decoupling: inhibition of return is observed at levels of saccadic control that are not normally associated with C^∗^ (Posner et al., 1995); conversely, it is *not* observed in parallel search, where perceptual “popout” is a fully conscious phenomenon (Treisman and Gelade, 1980). However, the control mechanisms underlying such autosuppressivity do not have to be conscious for them to form the basis of the experienced autosuppressivity, and in order to form its NSCP they only need to operate when the autosuppressivity occurs. As such, the neural bases for inhibition of return remain strong candidates for the NSCP of this aspect of C^∗^.
10. **Self-referentiality.** Computationally, it is not difficult to construct a computer program that includes itself as a component in its representation. Indeed, the representation of the external player as an element in the programmed domain is a common feature of computer games known as an “avatar.” Such an avatar escapes Russell’s Paradox by not being a full representation that actually contains itself, but only a reduced representation of the major features of itself in model form. It is not so clear how the neural implementation of an avatar could be achieved, but to do so is a further prerequisite of the NSCP. Note that this concept, of self-referentiality being a testable aspect of the NSCP while referentiality *per se* is not, is itself paradoxical. Self-referentiality can be tested by identifying a brain process that switches on and off concurrently with the switch between awareness of the self “avatar” and of other content, whereas referentiality cannot be tested because it is an unavoidable property of C^∗^, and there is no non-referential form of C^∗^ against which to test the “off” state of a candidate process.

## Discussion

### Functions of Consciousness

A further aspect of consciousness that can be considered is its **evolutionary function** (Bridgeman, 2011; Earl, 2014), as distinct from its neural integrative, adaptive and working memory functions, which are commonly highlighted (e.g., Baars et al., 2013). Indeed, many aspects of brain function are integrative, adaptive and mnemonic without passing the threshold of conscious awareness, such as the procedural memory functions of the cerebellum and basal ganglia. It is evident, therefore, that such brain functions do not require consciousness *per se*, and that such neural integrative, adaptive and mnemonic functions therefore do not require consciousness to operate.

Bridgeman (2011) argues that consciousness allows organisms to avoid the tyranny of response to the immediate (e.g., Pavlovian) environment, allowing the organism to superpose its goal-directed needs into the situational response. He concludes that such behavior requires the operation of working memory, and that consciousness is therefore a particular form of working memory. However, although **goal-directedness** may be a characteristic property of consciousness, it does not seem a sufficient criterion for the inference of consciousness as an **experience**. Virtually all behaving organisms engage in such goal-directed behavior in one form or another, but we would hesitate to ascribe consciousness to all forms of goal-directed behavior (such as cows eating grass, for example). Indeed, goal-directed behavior can be observed in single-celled micro-organisms, such as the hunting behavior of dinoflagellates and planaria, based on the information gleaned from their unitary subcellular eye (Schwab, 2012). Thus, behavioral goal-directness is a property – indeed, the essential property – of all behaving organisms, or animals, making it difficult to distinguish the role of goal-directedness in consciousness *per se* from that in behavior in general.

An alternative view of the role of consciousness in working memory is that it represents the **interface** of the memory storage process. There is substantial evidence that we can only remember items from the sensory world that were attended (i.e., that were a focus of conscious awareness; Penfield, 1958). Although unattended items may be processed in some form to allow their characterization as uninteresting targets for attention, through what is known as pre-attentive processing (Neisser, 1967), such items do not reach the site of accessible memory. Only attended items can be recalled from memory. It therefore seems that consciousness may represent the gateway to memory. While not all items that reach consciousness may be remembered, it seems to be the case that all items that are remembered must have reached consciousness.

Although consciousness is thus a *sine qua non* for laying down the memory for an item, it is nevertheless not required for the memory *per se*. Indeed, the very concept of memory implies a lack of consciousness, for the act of **remembering** corresponds precisely to bringing the item back into consciousness from its latent storage status outside of consciousness. This lack consciousness is evident for the vast range of items in long-term memory, such as the name of your first-grade teacher (which you may not have brought to consciousness for decades), but is also true for short-term iconic memory. We have all had the experience of being told a phone number, then doing competing activity during which we are not conscious of the number, then being able to recall the phone number by directing attention to the internal auditory “echo” of that number that is still available for a few minutes, though outside the immediate consciousness until it is accessed.

Equally, consciousness may be distinguished from the more interactive concept of **working memory** (Baddeley and Hitch, 1974), the earlier form of the global workspace that is currently being championed by Baars et al. (2013). There are three aspects to these forms of operation, which form the core operations of the process we call “thinking”: the recall of items from memory, the sequence of working operations on the items, and the consciousness of this process. Consider the quiz question of whether an item is bigger than a breadbox, for an item such as a rugby ball. We have to recall the item from memory, examine the memory to ascertain its dimensions, do likewise for the standard concept of a breadbox, compare the sets of dimensions (with appropriate rotation to the best fitting orientation) and make the decision as to which is larger. Indeed, we have to decide which form of breadbox is intended, the single-loaf kind that would be too small for the rugby ball or the multiloaf breadbin that would easily be large enough. Since each of these operations require the act of recall from memory, followed by operations on them, it seems to be a misnomer to call them a form of memory *per se*, even if it were an active form. The term “working memory” was perhaps a strategy to avoid the use of the term “consciousness” in the reductionist milieu of the mid-20th century, but including all these operations seems to inflate the storage function of memory to an implausible extent. It is preferable to restrict the term “memory” to the storage function of retaining the information after moving it outside the theater of consciousness.

Finally, what light does this analysis shed on the Baars’ **global workspace** as the essence of consciousness? In the breadbox quiz, we become conscious of posing the question of recalling the memory of the rugby ball, of its scale, of recalling the breadbox, of its relative scale, of aligning the two up for comparison, and of the decision. But we are not conscious of all these factors at the same time. At least at the beginning of the process, while we are recalling the shape of the rugby ball from those of other sports balls, we are not conscious of considering the type of breadbox. It is only when all the components have been recalled from memory that we may perhaps be conscious of them all together. So, while the global workspace may be the specific arena of the **operations** of consciousness, it does not seem to be an accurate characterization of the core function of consciousness *per se*. Consciousness seems to be better characterized as the role of operational attention *within* the global workspace, rather than the global workspace as a whole. In this sense, consciousness is conceptualizable as a “mental grasping” capability requisite for the manipulation of mental constructs within the global workspace.

In summary, the evolutionary function of consciousness may be not so much a mechanism to introduce goal-directed aspects into the control of behavior as one to function as the gatekeeper for memory storage, such that only aspects of the sensory input that pass the criterion for reaching consciousness can be stored in memory, while all other aspects are lost (Penfield, 1958). The stored memories themselves decay over time, so they may also tend to be lost eventually, but many are retained for long periods, or even a lifetime, especially those that were experienced with heightened consciousness. Thus, while “attention” describes the selective function of which aspects of the sensory input are the focus of the gatekeeping function, “consciousness” describes the activation level through which the elaborated sensory input becomes laid down as a memory trace, and reinforced or reorganized in memory when recalled through the working memory mechanism.

### Comment on Integrated Information Theory

Perhaps the most salient current analysis of consciousness is the Integrated Information Theory of Tononi (2008). The present analysis does not extend to a full evaluation of its claims, but it is relevant to address one its core axioms. This is the axiom that consciousness has a “rich conceptual structure composed of a very large number of concepts and relations” (Tononi et al., 2016, p. 457), which correspond to all the **phenomenal** distinctions that make up our reported conscious experience. To treat this property as axiomatic of consciousness seems to completely miss the point, however, since even a consciousness that is limited to very few concepts should still qualify as a valid form of phenomenal consciousness. Tononi’s specification is roughly equivalent to the multifacetedness property of consciousness in the present analysis, so to that extent we agree, but to treat it as an axiomatic defining property of consciousness seems misguided (compare Bayne, 2018). Even if the maximum capacity of consciousness was severely limited, as it presumably must be in the lowest level of organism that experiences it, that limitation does not detract from the fact of that consciousness. Indeed, it is a common experience that one’s consciousness becomes drasticaly limited in “conceptual structure” when one is very tired or otherwise debilitated, though it may still have the qualitative vividness that is the core characteristic of phenomenal consciousnees. Conversely, even if a complex system, a deep-learning computer or the Internet, develops a “very large number of concepts and relations,” that does not mean that it is conscious. In this sense IIT (Tononi, 2008; Koch et al., 2016; Tononi et al., 2016) cannot be considered to be a theory of phenomenal consciousness *per se*, though it could be considered to be a valid conceptualization for what is termed “access consciousness.”

## Conclusion

This article has had the goal of expanding the soup-to-nuts philosophy of Emergent Aspect Dualism to the experienced properties of consciousness, as one of the prime forms of emergence, and one to which the only access is subjective report. To extend the probing to the NSCP, a full specification of the properties of consciousness as subjectively experienced is provided in forms that are neuroscientifically testable. These properties are then considered against those of the global workspace and IIT conceptualizations of consciousness to highlight the differences between those viewpoints and the current framework, which is the explicit testability of consciousness conceived as the experiential focus of operational attention by which transient sensory input is converted to long-lasting memories.

## Author Contributions

The author confirms being the sole contributor of this work and has approved it for publication.

## Conflict of Interest

The author declares that the research was conducted in the absence of any commercial or financial relationships that could be construed as a potential conflict of interest.

## Footnotes

1 It is recognized that, although the Schrödinger energy equation is typically considered the basis of all physics, the only definition of energy in physics is the anemic “work done,” which specifies only the magnitude of the energy. Here, the paradigm concept of an energetic process is an energetic particle traveling through the universe, which only does work when absorbed.

2 The concept of consciousness as an emergent function of brain activity is widely accepted in neuroscience and has a long philosophical history, as summarized in the Stanford Encyclopedia of Philosophy https://plato.stanford.edu/archives/sum2015/entries/properties-emergent/.

3 The “you” in this context should be read as a stand-in for any conscious decision maker.

4 It may be noted that a non-quantized approach to the wave function in the form of the Einstein-Maxwell-Dirac classical field theory is under active development (e.g., Finster et al., 1999; Vegt, 2019).

5 Note that, since the original publication of the distinction of the neural substrate for conscious processing (NSCP; Tyler, 2015) from the neural correlate of consciousness (NCC; Crick and Koch, 1990; Koch, 2004), Tononi et al. (2016) changed their notation from the longstanding NCC terminology to the “physical substrate of consciousness (PSC)” (without providing any justification for the change).

6 It is assumed that such reports are obtained under fully non-coercive conditions, such that they may be taken on faith to be valid.

7 Note that such rapid eye movements are a good example of an NCC, but do not constitute an NRCP since no one would consider such movements of the eyes to be a plausible substrate of consciousness.
